# Bridging Gaps in Cancer Care: Utilizing Large Language Models for Accessible Dietary Recommendations

**DOI:** 10.3390/nu17071176

**Published:** 2025-03-28

**Authors:** Julia A. Logan, Sriya Sadhu, Cameo Hazlewood, Melissa Denton, Sara E. Burke, Christina A. Simone-Soule, Caroline Black, Corey Ciaverelli, Jacqueline Stulb, Hamidreza Nourzadeh, Yevgeniy Vinogradskiy, Amy Leader, Adam P. Dicker, Wookjin Choi, Nicole L. Simone

**Affiliations:** 1 Department of Radiation Oncology, Sidney Kimmel Medical College, Thomas Jefferson University, Philadelphia, PA 19107, USAsriya.sadhu@students.jefferson.edu (S.S.); sara.burke@jefferson.edu (S.E.B.); adam.dicker@jefferson.edu (A.P.D.); wookjin.choi@jefferson.edu (W.C.); 2 Sidney Kimmel Comprehensive Cancer Center, Thomas Jefferson University Hospitals, Philadelphia, PA 19107, USA; 3 Department of Medical Oncology, Sidney Kimmel Medical College, Thomas Jefferson University, Philadelphia, PA 19107, USA

**Keywords:** artificial intelligence, breast cancer, weight management, cancer, diet

## Abstract

**Background/Objectives**: Weight management is directly linked to cancer recurrence and survival, but unfortunately, nutritional oncology counseling is not typically covered by insurance, creating a disparity for patients without nutritional education and food access. Novel ways of imparting personalized nutrition advice are needed to address this issue. Large language models (LLMs) offer a promising path toward tailoring dietary advice to individual patients. This study aimed to assess the capacity of LLMs to offer personalized dietary advice to patients with breast cancer. **Methods**: Thirty-one prompt templates were designed to evaluate dietary recommendations generated by ChatGPT and Gemini with variations within eight categorical variables: cancer stage, comorbidity, location, culture, age, dietary guideline, budget, and store. Seven prompts were selected for four board-certified oncology dietitians to also respond to. Responses were evaluated based on nutritional content and qualitative observations. A quantitative comparison of the calories and macronutrients of the LLM- and dietitian-generated meal plans via the Acceptable Macronutrient Distribution Ranges and United States Department of Agriculture’s estimated calorie needs was performed. **Conclusions**: The LLMs generated personalized grocery lists and meal plans adapting to location, culture, and budget but not age, disease stage, comorbidities, or dietary guidelines. Gemini provided more comprehensive responses, including visuals and specific prices. While the dietitian-generated diets offered more adherent total daily calorie contents to the United States Department of Agriculture’s estimated calorie needs, ChatGPT and Gemini offered more adherent macronutrient ratios to the Acceptable Macronutrient Distribution Range. Overall, the meal plans were not significantly different between the LLMs and dietitians. LLMs can provide personalized dietary advice to cancer patients who may lack access to this care. Grocery lists and meal plans generated by LLMs are applicable to patients with variable food access, socioeconomic means, and cultural preferences and can be a tool to increase health equity.

## 1. Introduction

Numerous studies have demonstrated the significant impact of dietary habits and weight management on the overall well-being of cancer patients, with a direct correlation between weight and prognosis [1,2]. This is particularly true in hormonally responsive cancers such as prostate and breast cancer [1,2]. Maintaining a healthy weight after a cancer diagnosis is complex. Many cancer patients have notable weight gain in the first year which is associated with poor outcomes [3]. The weight gain is multifactorial and, in part, related to the stress of treatment, the use of steroids to minimize the toxicity of chemotherapy, and other therapies that are known to slow patients’ metabolism such as hormone therapy [4,5]. Adhering to a proper nutritional plan can improve treatment response while minimizing toxicity, reducing recurrence risk, and improving survival [6,7]. However, despite the growing body of evidence supporting weight management, many patients lack access to adequate nutritional counseling and support [8]. The integration of dietary interventions into oncology care remains inconsistent and underutilized, in part due to systemic barriers such as insurance limitations, resource availability, and healthcare infrastructure constraints [8].

Significant barriers to weight management are the knowledge of patients and lack of counseling by oncologists or dietitians. Nutritional knowledge among the general public varies widely, often influenced by conflicting information in the media, diet trends, and misinformation, making it difficult for cancer patients to discern what is truly beneficial for their health [9]. Despite the importance of proper nutrition in cancer care, physicians often lack the time to provide dietary counseling during clinical visits, and medical school curricula dedicate minimal time to nutrition education, leaving many doctors without the necessary background to offer detailed, evidence-based dietary guidance [10]. Unlike other aspects of cancer treatments such as chemotherapy, radiation, and surgical interventions, dietary counseling is often considered an “ancillary service” rather than essential, despite its benefits for patient outcomes [11]. This lack of emphasis has led to significant gaps in patient access to reliable nutritional support. In fact, nutritional counseling is not typically covered by insurance for outpatient oncology patients which creates disparities in access to personalized dietary guidance [12]. Patients who seek professional nutritional advice either rely on larger cancer centers to provide dietician services to their patients or must pay out of pocket [11]. It is also possible that recommendations given may not be tailored to their specific medical conditions, treatment regimens, or dietary restrictions.

One significant challenge of integrating nutrition into cancer care is limited access to individualized dietary counseling, primarily driven by a shortage of oncology-registered dietitians as well as structural constraints within oncology care [13]. Even when dietitians are available, long wait times, limited insurance coverage, and inconsistent follow-up often prevent patients from receiving the ongoing support necessary to implement sustainable dietary changes. Unlike chemotherapy, radiation, and surgery—where standardized protocols ensure consistency in treatment—oncology nutrition lacks universal guidelines, which leads to variability in recommendations between dietitians leaving patients uncertain about which dietary strategies to follow [14]. This is further compounded by oncology visits that prioritize treatment discussions, symptom management, and urgent medical decisions, leaving little room for in-depth dietary counseling. With the rising prevalence of cancer worldwide, there are simply not enough dietitians to provide individualized counseling to every patient or offer consistent follow-up visits as patients attempt to implement dietary changes in real time [13]. Even in institutions with dedicated oncology dietitians, patients may receive only a single consultation or generalized dietary recommendations, not the comprehensive, personalized support necessary for long-term adherence and meaningful dietary change.

Even where dietary guidance is provided, many patients struggle to implement recommendations due to financial, geographic, and cultural barriers [15]. The cost of specialized dietary interventions, such as high-protein supplements or organic foods, can be prohibitive, particularly for anyone facing financial hardship. Patients living in food deserts—areas with limited access to affordable, nutritious food—may be unable to obtain the ingredients necessary for a balanced diet [8]. Additionally, patients with low socioeconomic status may lack the resources, time, or cooking facilities to prepare recommended meals, further widening disparities in access to proper nutrition. Cultural dietary preferences and language barriers further complicate nutrition care, as many standardized dietary recommendations do not account for diverse food traditions [16]. Patients from different cultural backgrounds may struggle to find suitable substitutions within their traditional diets, making adherence to dietary recommendations challenging. Furthermore, language barriers may prevent patients from fully understanding dietary guidance, leading to misinterpretations or difficulty implementing suggested changes [17]. Without individually tailored, culturally competent nutritional counseling, many cancer patients, especially those from underserved populations, may miss out on the benefits of dietary interventions, underscoring the need for more accessible, adaptable, and inclusive approaches to nutrition in cancer care.

Given these challenges, there is an urgent need for alternative methods to provide cancer patients with accessible, personalized dietary guidance. Over the past decade, advances in artificial intelligence and natural language processing have led to the widespread availability of large language models (LLMs), which have been increasingly integrated into various aspects of healthcare [18]. LLMs such as ChatGPT and Gemini have demonstrated the ability to generate personalized, context-specific information, making them a promising tool for delivering tailored dietary recommendations to cancer patients. These models are already being used in medical communication, patient education, research, and administrative tasks, suggesting their potential utility in providing nutritional counseling for oncology patients [19,20]. They also offer large scalability, 24/7 availability, personalization, near-instantaneous information retrieval, and a vast potential for future advancement.

In this study, we sought to evaluate the ability of LLMs to provide individually tailored dietary recommendations for patients with breast cancer. Specifically, we analyzed the capacity of ChatGPT and Gemini to tailor meal plans and grocery lists based on cancer stage, comorbidities, geographic location, cultural preferences, age, dietary guidelines, budget constraints, and store availability. Given the disparities in access to traditional oncology nutrition services, LLMs may represent a scalable, cost-effective solution to provide equitable dietary support for cancer patients. By assessing the accuracy, adaptability, and comprehensiveness of LLM-generated dietary advice, we aim to determine whether these tools can bridge gaps in nutrition care, improve patient outcomes, and promote health equity in oncology.

## 2. Materials and Methods

**Figure 1:**
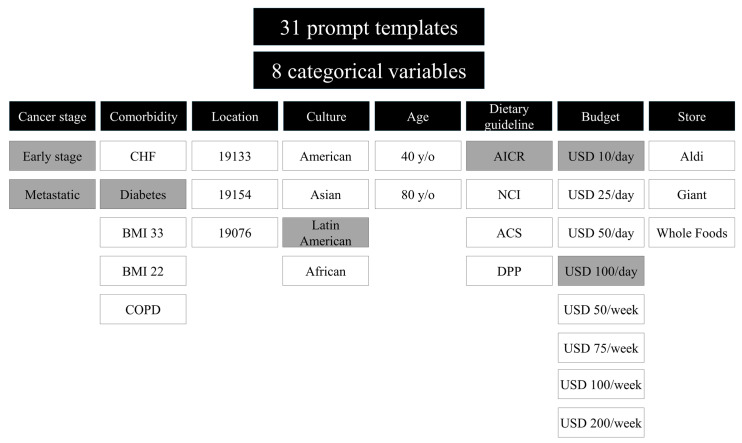
A schematic of LLM prompts designed to evaluate the dietary recommendations generated by ChatGPT and Gemini. A total of 31 zero-shot prompt templates with prompt variations within 8 categorical variables, including cancer stage, comorbidity, location, culture, age, dietary guideline, budget, and store, are shown. One variable was changed in each prompt. Seven of these prompts were selected (highlighted in gray) and four dietitians also responded to them.

To evaluate the feasibility of LLMs’ ability to provide dietary suggestions for cancer patients, we designed 31 zero-shot prompt templates aimed at eliciting meal plans and grocery lists tailored to breast cancer patients. The zero-shot prompts were used to assess how well the models could generate plans without prior training which could ultimately increase future generalizability. These templates were structured to generate personalized dietary recommendations that could be adapted based on disease type, nutritional needs, and individual preferences. The prompts were carefully constructed to explore variations across eight categorical variables: cancer stage, comorbidities, zip code (geographic location), cultural dietary preferences, age, adherence to specific dietary guidelines, budget constraints, and available grocery stores. These variables were selected by the oncologist and dieticians after careful consideration of demographic information and trends seen in patient care settings while including variables and barriers to achieving a healthy diet. By methodically modifying these variables, we sought to analyze the flexibility and accuracy of LLM-generated dietary recommendations in addressing diverse patient needs (Figure 1).

The prompts were input into two AI models: GPT-3.5 (OpenAI) [21] and Gemini (Google AI, Version 1.0) [22]. It is important to acknowledge that while this analysis was conducted using older LLMs, the findings remain consistent and relevant. The models were queried using a standardized prompt format: “Develop an optimal grocery list and meal plan for one day for a breast cancer patient who needs to eat a healthy diet”. Each of the categorical variables was systematically introduced into this template to assess how well the models adapted their recommendations. To minimize potential model learning and bias, new sessions were frequently started, and ChatGPT (GPT-3.5) was used without a logged-in user, for the purpose of preventing persistent memory effects. Each prompt was tested only once to ensure that responses were generated independently and without iterative refinement.

In order to compare the LLM-generated responses with actual expert recommendations, four board-certified oncology dietitians from Jefferson Oncology were provided with seven selected prompts, matching the seven prompts used for the AI models, and were asked to provide their own dietary recommendations (Figure 1). The prompts were chosen after an initial qualitative assessment of the LLM responses, which led to the exclusion of location, age, and store variables due to their limited impact on dietary formulation. The goal was to compare the depth, adherence, and personalization of the LLM responses against the meal planning provided by the dietary experts. The dietitians did not have access to the LLM-generated plans when crafting their responses. After developing their recommendations, the AI-generated meal plans were reviewed to ensure accuracy, clinical appropriateness, and alignment with oncology nutrition guidelines.

The nutritional content of both the LLM- and the dietitian-generated meal plans was quantified using the United States Department of Agriculture (USDA) FoodData Central database [23]. Calories, fat, protein, and carbohydrate values were calculated for each meal plan and analyzed for adherence to the Acceptable Macronutrient Distribution Ranges (AMDR). The AMDR recommends 20–30% of daily calories from fat, 10–35% from protein, and 45–65% from carbohydrates. In addition, total calorie recommendations were compared to the USDA’s estimated daily energy needs for women aged 40 and older, accounting for all activity levels (1600–2200 kcal) [24].

**Figure 2:**
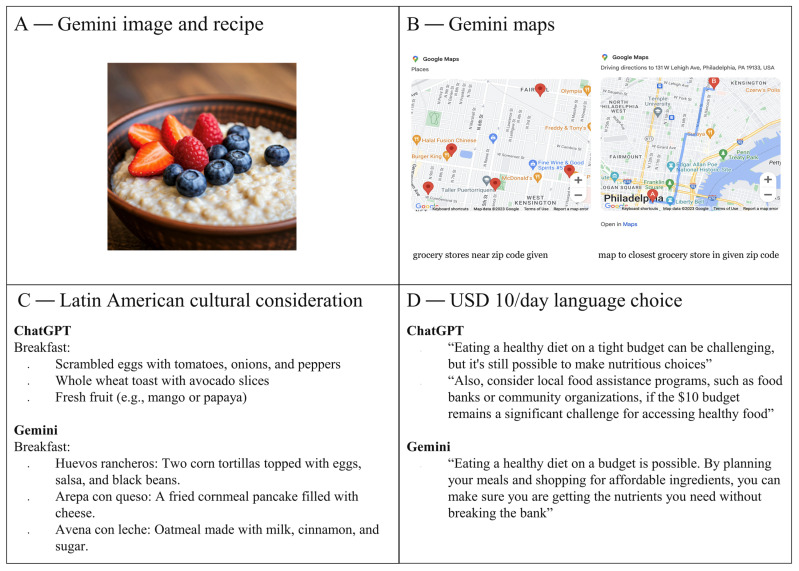
Qualitative observations of ChatGPT’s and Gemini’s responses. (**A**) Gemini provided photos with linked recipes for some meal plans. Panel (**A**) is an example of a Gemini-generated image comparable to the one provided, for copyright purposes. ChatGPT did not provide any photos or recipe links. (**B**) A map from Gemini showing all nearby grocery stores to the zip code specified and another map giving directions to the nearest grocery store. (**C**) A comparison of breakfast suggestions for Latin American cuisine between the two LLMs. (**D**) The response of both LLMs to the request for a budget of USD 10 for a day. ChatGPT used language such as “tight budget”, suggesting “food assistance programs”, while Gemini simply stated that it is possible to achieve a healthy diet.

Statistical analysis was conducted to determine significant differences between the average LLM-generated meal plan and the average dietitian-generated meal plan for each given prompt using *t*-tests. Beyond numerical differences, broader patterns in the LLM responses were identified and compared to the plans designed by the dietitians. Each was assessed for aspects such as variability, cultural adaptability, specificity, and adherence to dietary guidelines. Key trends and discrepancies between the LLM responses and expert dietary recommendations were recorded to evaluate the potential role of LLMs in supporting nutritional counseling for cancer patients (Figure 2).

## 3. Results

All prompts provided to the LLMs resulted in dietary recommendations that consisted of meal plans and grocery lists. The meal plans were designed to fit within various constraints, including budget, calorie requirements, and cultural preferences. It was noted that Gemini added several more points of reference than ChatGPT and included items such as photos, maps, and even meal titles in Spanish when Latin American cuisine was requested. Furthermore, Gemini incorporated economically sensitive terms when responding to requests for low-budget meal plans (Figure 2). These responses reflected a greater sensitivity to economic constraints, making it more accessible for individuals with limited resources. On the other hand, ChatGPT was generally more focused on meal preparation and ingredient suggestions without extending as much context, such as cultural specificity or visual aids. Both Gemini and ChatGPT were able to design meal plans with maximum dollar constraints and offered flexibility in budget allocation (e.g., USD 10–100 for one day, USD 50–200 for one week). Each LLM provided grocery lists that outlined the cost of individual ingredients, further demonstrating an ability to cater to specific financial needs with an example noted in Supplemental Data S1.

The output from Gemini provided exact prices in response to prompts that specified a grocery store, helping to ensure that users had precise information on ingredient costs, thereby making it easier to plan within individual budget constraints. ChatGPT, on the other hand, included specific store brands and sale suggestions but omitted precise prices. This subtle difference in approach showed that Gemini’s more detailed, store-specific recommendations would be particularly useful in regions with high variability in food prices. Both LLMs adjusted their recommendations based on prompts requesting meals from different cuisines, including Asian, African, and Latin American foods. Interestingly, American cuisine appeared to be the default choice for both models when no specific cuisine was requested, likely reflecting the models’ training data biases or a more general, culturally neutral approach.

There were no notable qualitative differences in the dietary recommendations generated by either LLM when the prompts were varied by age, cancer stage, comorbidities, or nutritional guidelines. This seems to suggest that both systems, while capable of providing general dietary advice, may not yet be able to fully tailor their responses to the unique dietary needs of individuals with specific health conditions. To ensure the quality of these responses, all LLM-generated plans were reviewed by a board-certified dietitian. The dietitian confirmed there were no glaring issues with the recommendations but did note there were recommendations for elevated animal protein in some of the meal plans as well as Gemini’s recommendation to avoid cruciferous vegetables for patients with COPD. This last point highlights a limitation in the LLMs’ ability to fully account for and adapt responses based on health-specific nuances, something which a human expert would be more likely to include based on clinical knowledge.

In order to compare the dietitian-provided plans with the LLM-generated plans, daily calorie content and the percentage of calories from macronutrients were quantified using the USDA FoodData Central database. This information was then compared to the USDA’s estimated calorie needs and Acceptable Macronutrient Distribution Range (AMDR) guidelines to evaluate the accuracy and nutritional balance of the meal plans. The quantifications of each of the four dietitian-provided meal plans were averaged for each prompt, and it was found that a higher number of their responses adhered to USDA’s estimated calorie needs in comparison to the plans generated by each LLM (Table 1). This is an indication that while both LLMs were capable of generating nutritionally balanced meal plans, the human experts were more consistent in meeting the specific caloric requirements for different individuals.

In terms of macronutrient distribution, both the dietitian and LLM diets adhered equally to the AMDR guidelines for the percentage of daily calories from protein (Supplemental Data S2). The LLM-suggested diets, however, were generally more adherent to the AMDR guidelines for the percentage of daily calories from fat and carbohydrates when compared to the average of the four dietitian responses, suggesting that the LLMs may be better at adjusting for specific fat and carbohydrate percentages. In general, the LLMs and the dietitians both recommended more meal plans with higher daily fat percentages and lower carbohydrate percentages than the AMDR. Detailed comparisons between the dietitian-provided versus AI-generated responses revealed that fat content for the USD 100/day diet (*p* = 0.028) and protein content for the USD 10/day diet (*p* = 0.009) were significantly different between the two groups. These findings indicate that while the overall meal plans from the LLMs were similar to those recommended by dietitians, specific dietary components like fat and protein were sometimes allocated differently. Aside from these significant differences, no other major differences were observed between the groups, suggesting that both approaches were largely aligned in terms of their overall dietary recommendations.

## 4. Discussion

In this study, we demonstrated that LLMs such as ChatGPT and Gemini could generate personalized dietary recommendations that aligned with dietitian-recommended calorie and macronutrient guidelines. The generated meal plans were successfully adapted to various constraints, including budget, cultural preferences, and grocery store availability, providing a degree of flexibility that is crucial for cancer patients with diverse needs. Notably, Gemini offered greater specificity by incorporating exact pricing and culturally relevant meal plans, whereas ChatGPT provided more general ingredient-based recommendations. While the LLM-generated plans were consistent with dietitian recommendations in terms of macronutrient balance, they were less precise in adjusting for factors such as comorbidities, cancer burden, and individualized calorie needs. We suspect that the LLMs may have outperformed the dietitians regarding the macronutrient ratios because the LLMs can quickly access large data sets associated with specific diets and reproduce this in prompt responses. Given the human nature of dietitians, the process to conduct this is much more tedious and prone to human error. Nevertheless, our findings indicate that LLMs have significant potential to improve dietary accessibility and nutritional support for cancer patients, particularly those who may lack access to specialized dietitians [25].

The ability of LLMs to generate meal plans tailored to socioeconomic and cultural considerations is one of the most promising aspects of this approach. Many cancer patients face financial burdens that limit their ability to adhere to dietary recommendations, and the ability to specify budget constraints ensures that meal plans are not only nutritionally sound but also financially feasible. Additionally, culturally appropriate meal plans are essential for dietary adherence, as patients are more likely to follow recommendations when they align with their food preferences and traditions. Gemini’s ability to calculate exact costs and integrate cultural adaptations, such as providing meal titles in Spanish when prompted for Latin American cuisine, demonstrates the utility of AI-driven dietary support in addressing disparities related to food access and cultural preferences [26]. Given that compliance with nutritional recommendations depends on accessibility, affordability, and cultural relevance, LLMs provide a valuable tool for improving patient adherence and, potentially, health outcomes [27]. However, both LLMs defaulted to American food, which indicates possible biases in training data. This should be further investigated in future studies. While the LLMs encountered difficulties in personalizing dietary modifications for morbidities and disease development, the use of further training would expand the capability of the LLMs to provide options adjusted for these variables.

Despite these advantages, oversight remains crucial to ensure the reliability of AI-generated dietary advice. Although LLMs can provide guidance when access to registered dietitians is limited, they cannot fully replace the expertise of trained professionals. Nutritional counseling is often complex, requiring adjustments based on a patient’s cancer type, treatment regimen, metabolic status, and comorbidities. Furthermore, dietitians are able to utilize strategies such as motivational interviewing and behavioral counseling, which LLMs lack. The shortage of registered dietitians trained in oncology already presents a barrier to care, with many patients experiencing long wait times, limited insurance coverage, or receiving only a single consultation instead of ongoing support. Additionally, there is no universally standardized approach to oncology nutrition, leading to variation in recommendations among dietitians themselves. While LLMs can help bridge this gap by offering immediate, evidence-based dietary advice, integrating AI-driven recommendations with professional dietetic oversight is essential to maintaining accuracy and ensuring patient safety [28]. Ensuring oversight will help to avoid inappropriate advice that may expose health professionals to legal prosecution.

There are limitations to consider when implementing LLM-based dietary recommendations. First, while LLMs provide general nutritional guidance, they do not yet account for individual physiological differences such as metabolism, activity level, or precise caloric needs for breast cancer patients undergoing treatment. Zero-shot prompts were used here to investigate the utility of LLMs without training; however, future studies should work with few-shot prompts or even fine-tune an OpenAI model that can be deployed as an application programming interface. Creating an application programming interface will allow the more generalizable use of LLMs for dietary guidance for all oncology patients. This study may be more generalizable to a broader cancer community but its utility is currently limited to breast cancer patients because each prompt specified this population. Additionally, discrepancies in nutritional databases and food brand variations may lead to slight inaccuracies in meal planning. Our study relied on USDA FoodData Central nutrient quantification, which may not fully capture variations in nutrient content across different food brands or international dietary standards. The study design was also somewhat limited with a small sample size of dietitians. Future studies with increased expert contributions may further solidify the study findings and reveal new intricacies. Furthermore, LLMs can produce “hallucinations” or generate inaccurate responses, particularly when prompted with highly specific or nuanced medical questions. Previous research has shown that ChatGPT-4 performs significantly better than ChatGPT-3.5 [29]. We also acknowledge the use of Gemini 1.0, which has since been updated. There is also potential for data bias in LLM training. The ethical considerations of LLMs in healthcare also need to be considered. There are concerns about privacy and data security as well as accuracy and the possible dehumanization of patient care. As LLMs continue to evolve, the further refinement of their training data and validation against established clinical guidelines will be necessary to improve their reliability in clinical practice [30].

The integration of LLM-generated dietary guidance into cancer care represents a significant step toward expanding access to evidence-based nutrition recommendations for all patients, regardless of financial, logistical, or social constraints. Nutrition plays a vital role in cancer outcomes, yet many patients struggle to implement dietary changes due to financial limitations, time constraints, and competing responsibilities such as employment or caregiving. The ability of LLMs to provide immediate, affordable, and personalized meal plans offers a scalable solution that aligns with the realities of modern life. By generating recommendations that are cost-conscious, culturally adaptable, and time-efficient, LLMs empower patients to make meaningful dietary changes that may positively influence their treatment response and overall prognosis. Given the well-established link between weight management and cancer outcomes, ensuring that all patients have access to actionable, personalized dietary advice is crucial. The widespread availability of LLMs makes this approach not only feasible but also transformative, helping to bridge longstanding gaps in oncology nutrition and offering a tool that has the potential to improve adherence, enhance quality of life, and ultimately impact survival.

## 5. Conclusions

Large language models (LLMs) like ChatGPT and Gemini demonstrate significant potential in expanding access to personalized dietary recommendations for breast cancer patients, particularly those facing financial, logistical, or cultural barriers to nutritional counseling. While LLM-generated meal plans effectively adapt to factors such as budget, location, and cultural preferences, their limitations in addressing individualized medical needs, comorbidities, and precise caloric requirements highlight the necessity of professional oversight. Despite these challenges, LLMs provide an accessible and scalable solution to improve dietary adherence, bridging disparities in oncology nutrition. Future efforts should focus on refining LLM training, integrating clinical validation, and developing AI-assisted tools that complement dietitian expertise, ensuring both accuracy and equity in cancer care.

## Figures and Tables

**Table 1:** Percentage of daily calories from macronutrients for different meal plans.

Variables	Macronutrients	Gemini	ChatGPT	Dietitian
Early Stage	Percentage of daily calories from fat	47.1%	36.3%	41.5%
	Percentage of daily calories from protein	23.8%	24.3%	21.1%
	Percentage of daily calories from carbohydrates	31.0%	40.9%	39.2%
Metastatic	Percentage of daily calories from fat	37.92%	42.43%	39.63%
	Percentage of daily calories from protein	19.2%	22.9%	21.2%
	Percentage of daily calories from carbohydrates	45.6%	35.8%	32.8%
USD 10/day	Percentage of daily calories from fat	31.6%	20.0%	38.7%
	Percentage of daily calories from protein	21.9%	22.0%	20.9%
	Percentage of daily calories from carbohydrates	47.1%	58.5%	40.3%
USD 100/day	Percentage of daily calories from fat	37.0%	19.1%	47.3%
	Percentage of daily calories from protein	20.9%	25.1%	21.5%
	Percentage of daily calories from carbohydrates	45.9%	56.3%	34.0%
Diabetes	Percentage of daily calories from fat	38.4%	49.2%	47.8%
	Percentage of daily calories from protein	22.0%	22.7%	22.8%
	Percentage of daily calories from carbohydrates	40.4%	30.7%	31.7%
Latin American	Percentage of daily calories from fat	35.6%	35.6%	41.5%
	Percentage of daily calories from protein	25.5%	29.8%	18.4%
	Percentage of daily calories from carbohydrates	37.5%	40.6%	41.0%
AICR	Percentage of daily calories from fat	41.5%	37.4%	42.7%
	Percentage of daily calories from protein	22.7%	23.5%	18.7%
	Percentage of daily calories from carbohydrates	37.5%	40.6%	41.0%

Percentage of daily calories from fat, protein, and carbohydrates in the suggested meal plans for the specified variables. Shaded cells adhere to the AMDR guidelines.

## Data Availability

The raw data supporting the conclusions of this are in the Supplemental Data S2.

## Supplementary Materials

The following supporting information can be downloaded at: https://www.mdpi.com/article/10.3390/nu17071176/s1.

## Abbreviations

The following abbreviations are used in this manuscript:

LLM	Large language model
USDA	United States Department of Agriculture
AMDR	Acceptable Macronutrient Distribution Range
