# Supplementary material for: Bridging Gaps in Cancer Care: Utilizing Large Language Models for Accessible Dietary Recommendations
Source: Nutrients. 2025 Mar 28;17(7):1176. doi: 10.3390/nu17071176 (PMC11990115; doi:10.3390/nu17071176)
Supplement: Supplementary file 1 [file nutrients-17-01176-s001.zip › nutrients-3532523-supplementary.pdf]

Supplemental Data S1. Example of one prompt and LLM response.

Prompt: Develop an optimal grocery list and meal plan for one day for an early stage breast cancer patient who needs to eat a healthy diet

Response:
Here's a sample grocery list and meal plan for one day for an early-stage breast cancer patient who needs to eat a healthy diet:

Grocery List

Breakfast

1 cup plain Greek yogurt
1/2 cup mixed berries
1/4 cup granola

Lunch

2 cups mixed greens salad
3 ounces grilled salmon
1/2 avocado
1 tablespoon olive oil
1 teaspoon balsamic vinegar

Dinner

4 ounces baked chicken breast
1 cup roasted vegetables (broccoli, carrots, zucchini)
1 cup quinoa

Snacks

1 apple
1/2 cup almonds
1 cup carrot sticks with hummus

Meal Plan

Breakfast:

Enjoy a bowl of Greek yogurt topped with mixed berries and granola for a nutritious and protein-rich breakfast.

Lunch:

Prepare a salad with mixed greens, grilled salmon, avocado, olive oil, and balsamic vinegar for a light and balanced lunch.

Dinner:

Savor a wholesome dinner of baked chicken breast, roasted vegetables, and quinoa, providing a combination of lean protein, fiber, and essential vitamins.

Snacks:

Throughout the day, munch on an apple for fiber and vitamins, almonds for healthy fats and protein, and carrot sticks with hummus for a satisfying and nutritious snack.

Additional Tips:

- Choose whole grains over refined grains whenever possible.
- Opt for lean protein sources, such as fish, poultry, and beans.
- Fill your plate with plenty of fruits and vegetables.
- Limit unhealthy fats, such as saturated and trans fats.
- Moderate your intake of added sugars and processed foods.
- Drink plenty of water throughout the day.
- Remember, this is just a sample meal plan, and you may need to adjust it based on your individual needs and preferences. It's always a good idea to consult with a registered dietitian or other qualified healthcare professional to get personalized advice on nutrition and diet for early-stage breast cancer patients.

Supplemental Data S2. Data from macronutrient calorie scoring by dietician and AI tools.

**Dietician 1**	**early stage**	**early stage CAL**	**%**	**metastatic**	**metastatic CA**	**%**	**Diabetes**	**Diabetes CAL**	**%**

**kcal**	1,742.8	1,742.8	100.0%	1,495.5	1,495.5	100.0%	1,568.6	1,568.6	100.0%
**fat**	76.7	690.5	39.6%	68.3	614.8	41.1%	73.2	658.6	42.0%
**protein**	94.3	377.3	21.6%	86.6	346.3	23.2%	108.2	433.0	27.6%
**carbohydrates (tota**	169.0	675.8	38.8%	136.2	544.6	36.4%	130.0	519.8	33.1%

**Dietician 2**	**early stage**	**early stage CAL**	**%**	**metastatic**	**metastatic CA**	**%**	**Diabetes**	**Diabetes CAL**	**%**
**kcal**	2343.3	2343.3	100.0%	2343.3	2343.3	100.0%	2343.3	2343.3	100.0%
**fat**	107.3	965.8	41.2%	107.3	965.8	41.2%	107.3	965.8	41.2%
**protein**	110.9	443.7	18.9%	110.9	443.7	18.9%	110.9	443.7	18.9%
**carbohydrates (tota**	244.7	978.7	41.8%	244.7	978.7	41.8%	244.7	978.7	41.8%

**Dietician 3**	**early stage**	**early stage CAL**	**%**	**metastatic**	**metastatic CA**	**%**	**Diabetes**	**Diabetes CAL**	**%**
**kcal**	1962.8	1,962.8	100.0%	1380.9	1,380.9	100.0%	1625.9	1,625.9	100.0%
**fat**	87.0	782.6	39.9%	48.9	439.7	31.8%	101.5	913.5	56.2%
**protein**	131.8	527.3	26.9%	88.0	352.1	25.5%	91.2	364.6	22.4%
**carbohydrates (tota**	177.0	708.0	36.1%	156.3	625.4	45.3%	99.7	398.6	24.5%

Dietician 4	early stage	early stage CAL	%	metastatic	metastatic CA	%	Diabetes	Diabetes CAL	%
kcal	2,483.9	2,483.9	100.0%	2,830.5	2,830.5	100.0%	2,371.4	2,371.4	100.0%
fat	125.3	1,127.6	45.4%	139.5	1,255.9	44.4%	136.4	1,227.8	51.8%
protein	104.2	416.9	16.8%	123.2	492.8	17.4%	132.5	529.9	22.3%
carbohydrates (tota	249.8	999.3	40.2%	287.7	1,150.6	40.7%	163.3	653.3	27.6%

Gemini	early stage	early stage CAL	%	metastatic	metastatic CA	%	Diabetes	Diabetes CAL	%
kcal	1979.4	1,979.4	100.0%	1763.5	1,763.5	100.0%	1465.8	1,465.8	100.0%
fat	103.5	931.9	47.1%	74.3	668.7	37.9%	62.6	563.5	38.4%
protein	117.7	471.0	23.8%	84.9	339.5	19.2%	80.8	323.1	22.0%
carbohydrates (tota	153.3	613.4	31.0%	201.2	804.9	45.6%	148.1	592.5	40.4%

ChatGPT	early stage	early stage CAL	%	metastatic	metastatic CA	%	Diabetes	Diabetes CAL	%
kcal	1792.3	1,792.3	100.0%	2055.8	2,055.8	100.0%	1865.5	1,865.5	100.0%
fat	72.2	650.0	36.3%	96.9	872.2	42.4%	101.9	917.3	49.2%
protein	108.8	435.0	24.3%	117.8	471.3	22.9%	105.9	423.7	22.7%
carbohydrates (tota	183.4	733.8	40.9%	184.0	736.0	35.8%	143.0	571.8	30.7%

AMDR GUIDELINES
Fat: 20-35%kcal
Protein: 10-35%kcal
Carbs: 45-65%kcal

Dietician 1	Latin America	Latin American	%	AICR Guidelin	AICR CAL	%	$10 per day	$10 per day C	%	$100 per day	$100 per day CAL	%
kcal	1,684.8	1,684.8	100.0%	1,256.1	1,256.1	100.0%	1,557.8	1,557.8	100.0%	2,441.4	2,441.4	100.0%
fat	61.7	555.5	33.0%	43.7	393.6	31.3%	49.1	441.9	28.4%	142.6	1,283.6	52.6%
protein	80.6	322.5	19.1%	74.3	297.1	23.7%	92.2	368.6	23.7%	141.3	565.2	23.1%
carbohydrates (tota	204.9	819.5	48.6%	148.6	594.3	47.3%	191.6	766.3	49.2%	171.2	685.0	28.1%

Dietician 2	Latin America	Latin American	%	AICR Guidelin	AICR CAL	%	$10 per day	$10 per day C	%	$100 per day	$100 per day CAL	%
kcal	2513.0	2513.0	100.0%	2343.3	2343.3	100.0%	1897.6	1897.6	100.0%	2463.3	2463.3	100.0%
fat	125.2	1126.4	44.8%	107.3	965.8	41.2%	82.3	740.6	39.0%	118.3	1064.8	43.2%
protein	84.7	338.6	13.5%	110.9	443.7	18.9%	78.3	313.1	16.5%	112.4	449.7	18.3%
carbohydrates (tota	275.1	1100.6	43.8%	244.7	978.7	41.8%	200.4	801.8	42.3%	251.1	1004.3	40.8%

Dietician 3	Latin America	Latin American	%	AICR Guidelin	AICR CAL	%	$10 per day	$10 per day C	%	$100 per day	$100 per day CAL	%
kcal	1412.5	1,412.5	100.0%	2372.9	2,372.9	100.0%	2105.0	2105.0	100.0%	2943.8	2943.8	100.0%
fat	74.3	669.0	47.4%	124.2	1,117.4	47.1%	105.5	949.5	45.1%	144.3	1298.4	44.1%

**protein**	63.9	255.6	18.1%	102.8	411.4	17.3%	97.6	390.4	18.5%	177.9	711.5	24.2%
**carbohyd rates (tota**	125.0	499.9	35.4%	230.6	922.4	38.9%	196.9	787.5	37.4%	251.3	1005.1	34.1%

**Dietician 4**	**Latin America**	**Latin American**	**%**	**AICR Guidelin**	**AICR CAL**	**%**	**$10 per day**	**$10 per day C**	**%**	**$100 per day**	**$100 per day CAL**	**%**
**kcal**	1,825.9	1,825.9	100.0%	2,385.8	2,385.8	100.0%	1,301.3	1,301.3	100.0%	3,126.3	3,126.3	100.0%
**fat**	83.1	748.3	41.0%	135.3	1,217.8	51.0%	61.0	549.1	42.2%	170.8	1,536.8	49.2%
**protein**	104.9	419.8	23.0%	89.6	358.4	15.0%	80.3	321.3	24.7%	160.2	640.8	20.5%
**carbohyd rates (tota**	180.7	722.8	39.6%	215.7	862.9	36.2%	105.3	421.1	32.4%	257.5	1,030.2	33.0%

**Gemini**	**Latin America**	**Latin American**	**%**	**AICR Guidelin**	**AICR CAL**	**%**	**$10 per day**	**$10 per day C**	**%**	**$100 per day**	**$100 per day CAL**	**%**
**kcal**	1257.9	1,257.9	100.0%	1708.8	1,708.8	100.0%	930.2	930.2	100.0%	2338.2	2338.2	100.0%
**fat**	49.8	448.0	35.6%	78.9	709.7	41.5%	32.7	294.1	31.6%	96.0	864.1	37.0%
**protein**	80.3	321.1	25.5%	96.9	387.5	22.7%	50.9	203.5	21.9%	122.0	488.0	20.9%
**carbohyd rates (tota**	123.9	495.7	39.4%	160.2	640.7	37.5%	109.6	438.3	47.1%	268.5	1074.0	45.9%

**ChatGPT**	**Latin America**	**Latin American**	**%**	**AICR Guidelin**	**AICR CAL**	**%**	**$10 per day**	**$10 per day C**	**%**	**$100 per day**	**$100 per day CAL**	**%**
**kcal**	2505.2	2,505.2	100.0%	1613.9	1,613.9	100.0%	1023.8	1023.8	100.0%	2566.0	2566.0	100.0%

**fat**	99.1	891.5	35.6%	67.0	603.3	37.4%	22.7	204.5	20.0%	54.4	489.7	19.1%
**protein**	186.7	746.7	29.8%	94.6	378.6	23.5%	56.4	225.7	22.0%	160.7	642.9	25.1%
**carbohydrates (tota**	223.8	895.0	35.7%	163.7	654.9	40.6%	149.7	598.8	58.5%	361.3	1445.2	56.3%
